# Ectopic Pancreas in the Stomach Successfully Resected by Endoscopic Submucosal Dissection

**DOI:** 10.1155/2015/147927

**Published:** 2015-03-24

**Authors:** Masaya Iwamuro, Takao Tsuzuki, Shogen Ohya, Hiroyuki Okada, Takehiro Tanaka, Keisuke Hori, Masahide Kita, Seiji Kawano, Yoshiro Kawahara, Kazuhide Yamamoto

**Affiliations:** ^1^Department of Gastroenterology and Hepatology, Okayama University Graduate School of Medicine, Dentistry, and Pharmaceutical Sciences, Okayama 700-8558, Japan; ^2^Department of General Medicine, Okayama University Graduate School of Medicine, Dentistry, and Pharmaceutical Sciences, Okayama 700-8558, Japan; ^3^Kawaguchi Medical Clinic, Okayama 700-0913, Japan; ^4^Department of Endoscopy, Okayama University Hospital, Okayama 700-8558, Japan; ^5^Department of Pathology, Okayama University Hospital, Okayama 700-8558, Japan

## Abstract

A 32-year-old Japanese man presented with a gastric submucosal tumor. Esophagogastroduodenoscopy showed a sessile submucosal tumor measuring approximately 10 mm in diameter on the greater curvature of the gastric antrum. Endoscopic ultrasonography examination revealed a solid tumor with a diameter of 11.8 mm, which was located in the deep mucosal and submucosal layers. The internal echogenicity was homogenous and hypoechoic. Biopsy examinations were performed twice but were not diagnostic since only the intact mucosal layer was obtained. The patient was subsequently diagnosed with ectopic pancreas in the stomach by endoscopic submucosal dissection (ESD). This case underscores the usefulness of the ESD technique for the pathological diagnosis of gastric submucosal tumors.

## 1. Introduction

Submucosal tumors, also called subepithelial tumors, are sometimes found during esophagogastroduodenoscopy examinations. The reported incidence of submucosal tumors on esophagogastroduodenoscopy is approximately 0.4% [1, 2]. Submucosal tumors in the upper gastrointestinal tract include a wide range of histologic entities, for example, leiomyoma, lipoma, ectopic pancreas, lymphoma, gastrointestinal stromal tumor (GIST), neuroendocrine tumor, neurofibroma, and schwannoma [3]. The histologic diagnosis of these tumors is sometimes difficult, even by stacked (bite-on-bite) biopsies. Generally, asymptomatic patients with submucosal tumors <2 cm can be followed up by periodic endoscopic examinations [4, 5]. However, the endoscopic surveillance of such submucosal tumors involves issues regarding cost-effectiveness, patients' compliance, and their anxiety that the tumor may be malignant [4, 6].

We recently treated a patient with ectopic pancreas in the stomach. The patient presented with a submucosal tumor in the gastric antrum. The tumor was suspected to be ectopic pancreas, but the possibilities of neuroendocrine tumor or other histologic entities could not be excluded. A total biopsy by the endoscopic submucosal dissection (ESD) technique was performed, and the diagnosis of ectopic pancreas was made. The differential diagnosis of the tumor in this patient and the role of ESD for the diagnosis of submucosal tumors are discussed.

## 2. Case Presentation

**Figure 1:**
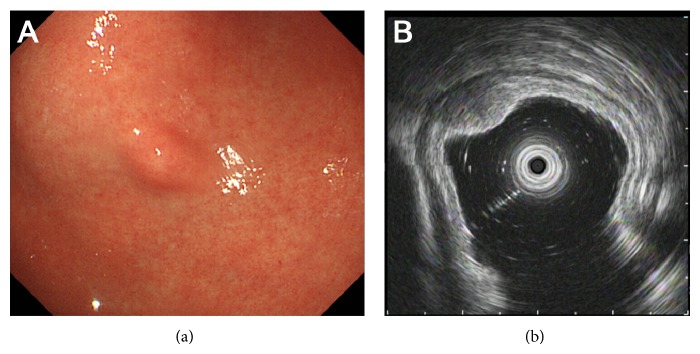
Endoscopic images. Esophagogastroduodenoscopy revealed a submucosal tumor with a diameter of approximately 10 mm in the greater curvature of the gastric antrum (a). Endoscopic ultrasonography showed a solid tumor with hypoechoic internal echogenicity measuring 11.8 mm, which was located in the deep mucosal and submucosal layers (b). Anechoic duct-like structure was not detected in the tumor.

A 32-year-old Japanese man underwent a barium swallow examination for the screening of esophagogastroduodenal diseases as part of a routine health checkup. Barium X-ray revealed a protruding lesion in the gastric antrum. The patient then underwent esophagogastroduodenoscopy at his family clinic, and it showed a sessile submucosal tumor measuring approximately 10 mm in diameter on the greater curvature of the gastric antrum (Figure 1(a)). The tumor had no apparent umbilication or central dimpling on the surface. There were no other lesions in the upper gastrointestinal tract except for fundic gland polyps. Atrophic changes were absent in the stomach. He was referred to our hospital for a further investigation of the gastric submucosal tumor.

The patient was currently healthy and had been taking no medications. A physical examination revealed no abnormalities. All laboratory findings were within the normal ranges. An endoscopic ultrasonography examination revealed a solid tumor with a diameter of 11.8 mm, which was located in the deep mucosal and submucosal layers (Figure 1(b)). The internal echogenicity was homogenous and hypoechoic, which was quite similar to the echogenicity of the deep submucosal layer. An anechoic duct-like structure was not detected in the tumor. A biopsy examination was performed, but the pathological examination was not diagnostic since only intact epithelium was obtained. Contrast-enhanced computed tomography scanning showed benign hemangiomas in the liver, whereas no lesion was detected in the stomach. Although the biopsy examination failed to provide a pathological diagnosis, ectopic pancreas in the stomach seemed most likely to be based on the location and endosonographic features. However, the possibility of a gastric neuroendocrine tumor (e.g., gastric carcinoid) could not be excluded.

**Figure 2:**
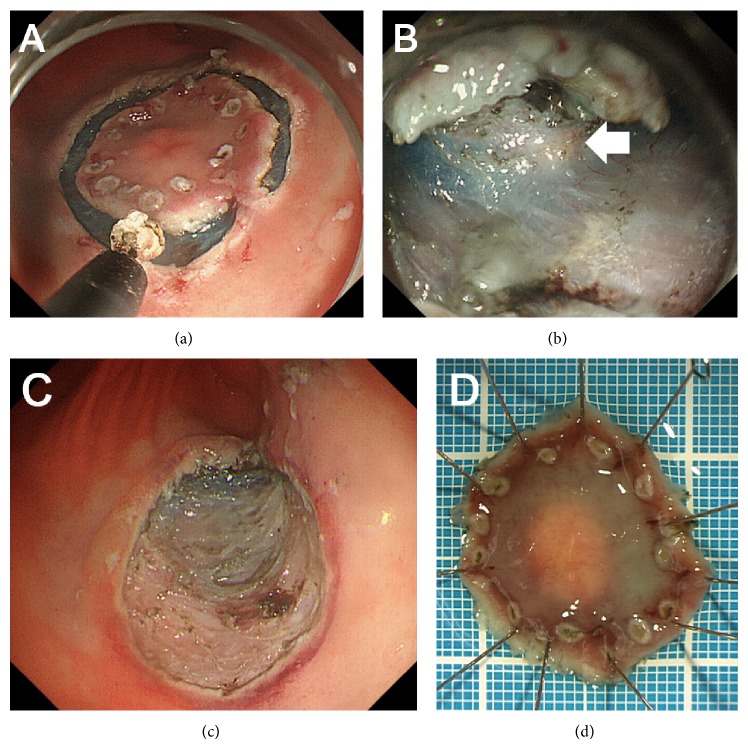
Images during the endoscopic submucosal dissection (ESD). After marking, a circumferential incision is made around the lesion (a). Whitish tumor tissue was seen within the submucosal layer (b, arrow). The tumor was completely resected by the ESD technique (c, d).

**Figure 3:**
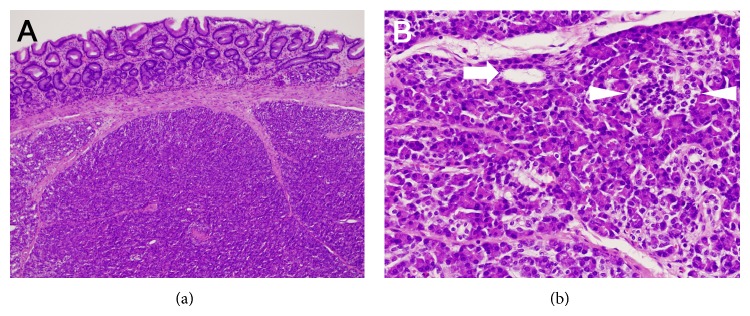
Pathological images. The tumor was located in the submucosal layer (a, hematoxylin and eosin [H&E] staining, ×4.2). Pancreatic tissues composed of acini, ducts (arrow), and islets of Langerhans (arrowheads) were seen in the tumor (b, H&E staining, ×20).

Additional esophagogastroduodenoscopy 6 months later showed that the gastric tumor was unchanged in size and shape. A biopsy examination was performed again but it was also nondiagnostic. At this point, we proposed the following two options to the patient as the follow-up/treatment strategy for his gastric tumor: to repeat esophagogastroduodenoscopy examinations annually unless the morphology had changed or to resect the tumor* en bloc* by the ESD technique as an excisional biopsy. He chose the latter option, and we therefore performed an ESD (Figure 2). After marking by using a dual knife (Olympus, Tokyo, Japan), 10% glycerin solution with 0.0025% epinephrine was injected in the submucosa. An initial incision was made by using the dual knife, and the tumor was dissected by using an insulation-tipped diathermic knife (IT Knife-2, Olympus). During dissection of the submucosal layer, the whitish tumor was visualized within the submucosal layer (Figure 2(b), arrow) and the tumor was successfully resected. The pathological examination revealed that the tumor was pancreatic tissues composed of acini, ducts, and islets of Langerhans (Figure 3). Consequently, a diagnosis of an ectopic pancreas of the stomach was made.

## 3. Discussion

Ectopic pancreas is pancreatic tissue located outside the pancreas without anatomic or vascular continuity with the normal pancreas. The prevalence of ectopic pancreas was reported to be 0.55%–13.7% in autopsy series [7, 8]. Since patients with ectopic pancreas are generally asymptomatic, most ectopic pancreas cases require no treatment. Conversely, surgical resection is required in rare instances, but it may cause complications such as hemorrhage [9], pancreatitis [10, 11], inflammatory change [12], obstructive jaundice, and malignant transformation [13]. In the stomach, the antrum is known as the most common site of ectopic pancreas.

Park et al. investigated the morphologic features of 26 patients with ectopic pancreas, and they observed umbilication or central dimpling in 34.6% (9/26 lesions) [14]. In endoscopic ultrasonography examination, 92.3% (24/26) of the lesions showed hypoechoic echogenicity, and 50.0% (13/26) were heterogeneous. Moreover, an anechoic duct-like structure was detected in 65.4% (17/26) of the tumors. On the basis of the sonographic layer of origin, 53.8% (14/26) of the tumors were found in the submucosal and proper muscle layers with or without extension into the subserosal or serosal layer, whereas the remaining 46.2% (12/26) of the tumors were located in the deep mucosal and/or submucosal layers. In the present patient, ectopic pancreas was the most likely diagnosis, because of the anatomic location and the hypoechoic echogenicity. However, the morphology and endoscopic ultrasonography findings for our patient's tumor were not conclusive for the diagnosis of ectopic pancreas, since it lacked umbilication, central dimpling, and duct-like anechoic architecture, which are defining characteristics of this disease [15].

The differential diagnoses of hypoechoic tumor located in the deep mucosal and submucosal layers as described in the present patient include neuroendocrine tumors. Neuroendocrine tumors are thought to arise from enterochromaffin-like cells that exist in the gastric glands of the gastric mucosa beneath the epithelium [16]. In endoscopic ultrasonography, these tumors are typically visualized as a homogeneous, mildly hypoechoic mass in the mucosal and submucosal layers [17]. For the pathological diagnosis of submucosal tumors, tissue sampling is essential. In the present patient, a stacked (bite-on-bite) biopsy was performed but adequate tissue was not obtained. Other procedures to take samples for pathological assessment include partial resection of the covering mucosa [18], endoscopic ultrasound-guided fine needle aspiration [19–21], and* en bloc* resection by an ESD technique. Since resection is recommended for gastric neuroendocrine tumors that are >10 mm because of the risk of lymph node metastasis [22], we proposed the option to resect the tumor* en bloc* by the ESD technique for a histologic diagnosis and treatment.

The usefulness of ESD for the pathological diagnosis of submucosal tumors has been reported by several groups [23–31]. Białek et al. performed ESD for 37 patients with submucosal tumors, and they noted that the overall rate of R0 resection was 81.1% (30/37 patients) [4]. Perforation occurred in two patients (2/37, 5.4%), bleeding in one (1/37, 2.7%), and pneumoperitoneum in three (3/37, 8.1%). Catalano et al. reported on the use of ESD for 20 patients with submucosal tumors; R0 resection was done in 90.0% of the patients (18/20). Though perforation occurred in three cases (15%), no patients experienced severe bleeding [30].

Although it has been thought that the* en bloc* resection of submucosal tumors originating from the proper muscle layer has an increased risk of perforation and bleeding [31], Białek et al. and Catalano et al. concluded that ESD is effective and relatively safe for resecting gastric submucosal tumors of proper muscle origin [4, 30]. On the other hand, submucosal tumors confined to submucosal layers are better indicated for* en bloc* resection by an ESD technique. Hoteya et al. resected nine gastric submucosal tumors of submucosal layer or mucosal muscle origin by ESD, and no complications were encountered [23]. Li et al. performed ESD for 24 patients with 29 lesions of neuroendocrine tumors [29], and though delayed bleeding occurred in one case (1/29, 3.4%), there were no procedure-related perforations. Consequently, resection by an ESD technique is a possible option for the pathological diagnosis of gastric submucosal tumors, particularly those which originate in the submucosal layer.

The use of ESD in the treatment of ectopic pancreas has been reported by several authors [32–36]. In 2010, Ryu et al. first evaluated the therapeutic usefulness and safety of ESD in the treatment of gastric ectopic pancreas in four cases [32]. In 2013, Zhong et al. reported the largest single-center case series reported to date [33]. They performed ESD for 46 cases, and the* en bloc* resection rate was 97.8% (45/46). Therefore, we consider ESD as a treatment option for ectopic pancreas in the stomach, particularly for symptomatic cases or cases with atypical morphological features.

In conclusion, we treated a patient with ectopic pancreas in the stomach. A histologic diagnosis was made by ESD in our patient. Resection by an ESD technique is a viable option for the pathological diagnosis of gastric submucosal tumors.
